# Prediction and diagnosis of renal cell carcinoma using nuclear magnetic resonance-based serum metabolomics and self-organizing maps

**DOI:** 10.18632/oncotarget.10830

**Published:** 2016-07-24

**Authors:** Hong Zheng, Jiansong Ji, Liangcai Zhao, Minjiang Chen, An Shi, Linlin Pan, Yiran Huang, Huajie Zhang, Baijun Dong, Hongchang Gao

**Affiliations:** ^1^ School of Pharmaceutical Sciences, Wenzhou Medical University, Wenzhou, 325035, China; ^2^ Lishui Central Hospital, The Fifth Affiliated Hospital, Wenzhou Medical University, Lishui, 323000, China; ^3^ Department of Urology, Renji Hospital, School of Medicine, Shanghai Jiao Tong University, Shanghai, 200127, China

**Keywords:** artificial intelligence, early diagnosis, metabolome, metabolic recovery, precision medicine

## Abstract

Diagnosis of renal cell carcinoma (RCC) at an early stage is challenging, but it provides the best chance for cure. We aimed to develop a predictive diagnostic method for early-stage RCC based on a biomarker cluster using nuclear magnetic resonance (NMR)-based serum metabolomics and self-organizing maps (SOMs). We trained and validated the SOM model using serum metabolome data from 104 participants, including healthy individuals and early-stage RCC patients. To assess the predictive capability of the model, we analyzed an independent cohort of 22 subjects. We then used our method to evaluate changes in the metabolic patterns of 23 RCC patients before and after nephrectomy. A biomarker cluster of 7 metabolites (alanine, creatine, choline, isoleucine, lactate, leucine, and valine) was identified for the early diagnosis of RCC. The trained SOM model using a biomarker cluster was able to classify 22 test subjects into the appropriate categories. Following nephrectomy, all RCC patients were classified as healthy, which was indicative of metabolic recovery. But using a diagnostic criterion of 0.80, only 3 of the 23 subjects could not be confidently assessed as metabolically recovered after nephrectomy. We successfully followed-up 17 RCC patients for 8 years post-nephrectomy. Eleven of these patients who diagnosed as metabolic recovery remained healthy after 8 years. Our data suggest that a SOM model using a biomarker cluster from serum metabolome can accurately predict early RCC diagnosis and can be used to evaluate postoperative metabolic recovery.

## INTRODUCTION

Renal cell carcinoma (RCC) accounts for 2–3% of all adult malignancies and has a mortality rate greater than 40% [1]. The incidence of RCC (all stages) is increasing yearly [2]. Early diagnosis provides the greatest chance for cure. However, more than 30% of RCC patients have metastatic disease at the time of diagnosis. This can be attributed to the lack of symptoms typically associated with early-stage RCC [3]. Clinical symptoms such as pain, the presence of a mass, or hematuria are generally not sufficient for early diagnosis [4]. Additionally, radiological methods for RCC diagnosis such as ultrasound, computed tomography, magnetic resonance imaging, and positron emission tomography are not always accurate [5, 6]. Finally, renal biopsy and histological diagnosis are invasive and time-consuming. Therefore, the development of new diagnostic strategies is critical for the prevention and management of RCC.

Precision medicine is based on the premise that variations in genetics, lifestyle, and environment between individuals can be used for early diagnosis and personalized care, and has shown great potential for cancer diagnosis and treatment [7]. Omics-based approaches in which a comprehensive set of genes, proteins, or metabolites are measured can reveal biological phenotypes at omics levels. These technologies can significantly advance precision medicine [8]. Recently, omics-based methods have been used to predict and diagnose various cancers [9]. For example, genomics- and proteomics-based approaches have been shown to be important for RCC diagnosis and for predicting patient prognosis [10, 11]. Metabolomics is a relatively new approach in which all low molecular weight metabolites in biological samples are analyzed. This approach can provide valuable insight into metabolic changes that occur during disease processes (e.g., carcinogenesis). We previously used NMR-based metabolomics to analyze differences in the serum metabolome between RCC patients and healthy subjects [12]. We found that this approach could discriminate between RCC patients with and without metastases and before or after nephrectomy. More recently, differences in the plasma metabolome between RCC patients and healthy subjects were identified using a similar approach [13]. Finally, Lin et al. [14] reported that liquid chromatography-mass spectrometry (LC-MS)-based serum metabolomics could be used to diagnose and stage RCC.

Technologies for data-driven prediction and diagnosis, especially those based on ‘omics’ data, must be able to effectively extract essential information from large data sets. Machine learning is a branch of artificial intelligence that simulates human learning and classification processes [15]. In contrast to conventional statistical methods involving multivariate regression or correlation analysis, machine learning provides the opportunity to learn from past data and then use the knowledge to classify new data [15]. In this study, we aimed to develop a new tool for the prediction and diagnosis of early-stage RCC using NMR-based metabolomics and self-organizing maps (SOMs). We then used this method to predict and diagnose RCC in an additional group of blinded subjects. Finally, we evaluated change in metabolic patterns in RCC patients before and after nephrectomy, and examined patient quality of life 8 years post-nephrectomy.

## RESULTS

### Optimization of SOM architecture

**Figure 1:**
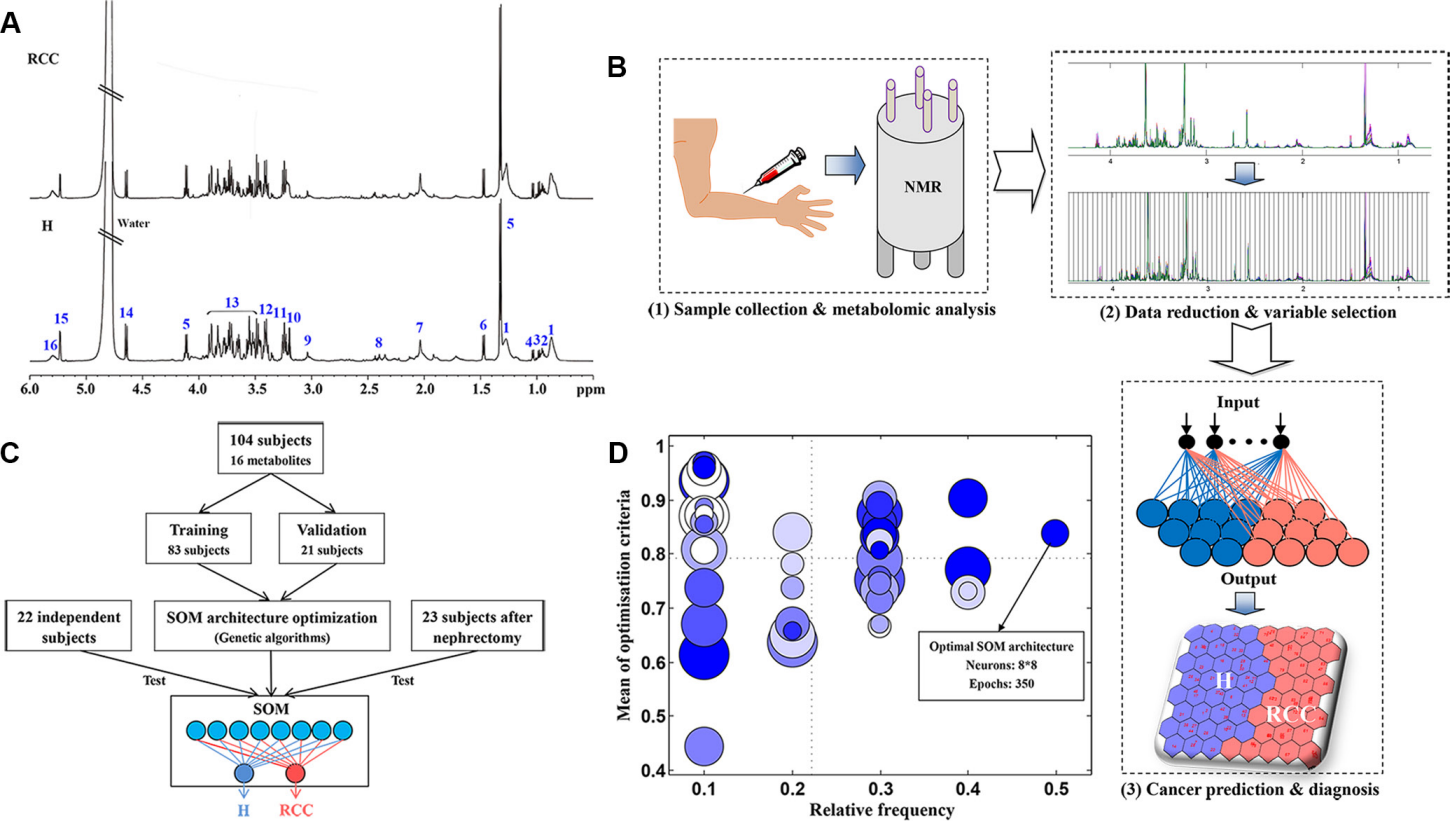
Development of the SOM model (**A**) The ^1^H NMR spectra from human serum samples used for the development of the SOM. The numbers correspond to the metabolites in Table S1; (**B**) The procedure for RCC prediction and diagnosis using the SOM: (1) sample collection and metabolomics analysis, (2) data reduction and variable selection, and (3) cancer prediction and diagnosis. (**C**) The development of the SOM. First, the SOM architecture was optimized using genetic algorithms. Second, the optimized SOM was trained and validated using 80% and 20% of the subjects, respectively. Finally, 22 independent subjects were analyzed to further evaluate the trained SOM model, and 23 additional subjects analyzed to evaluate metabolic patterns after nephrectomy. (**D**) The bubble plot of SOM architecture optimization by genetic algorithms. Each bubble represents a type of SOM architecture. The size and color of the bubbles are proportional to the number of neurons and epochs in the SOM, respectively.

The optimal results for the SOM architecture by genetic algorithm (GA) are shown in the bubble plot in Figure 1D. This plot demonstrates the relative frequency of selection in the GA and the mean value of the optimization criterion. Higher values for these two parameters were indicative of a better SOM architecture. Each bubble represents the SOM architecture, and the bubble size and color are proportional to the number of neurons and epochs, respectively. Considering model performance and structural complexity, a SOM architecture with 8 × 8 neurons and 350 epochs was selected as the optimal model for all metabolites, and 10 × 10 neurons and 50 epochs for a biomarker cluster.

### Identification of a biomarker cluster for the prediction and diagnosis of RCC

**Figure 2:**
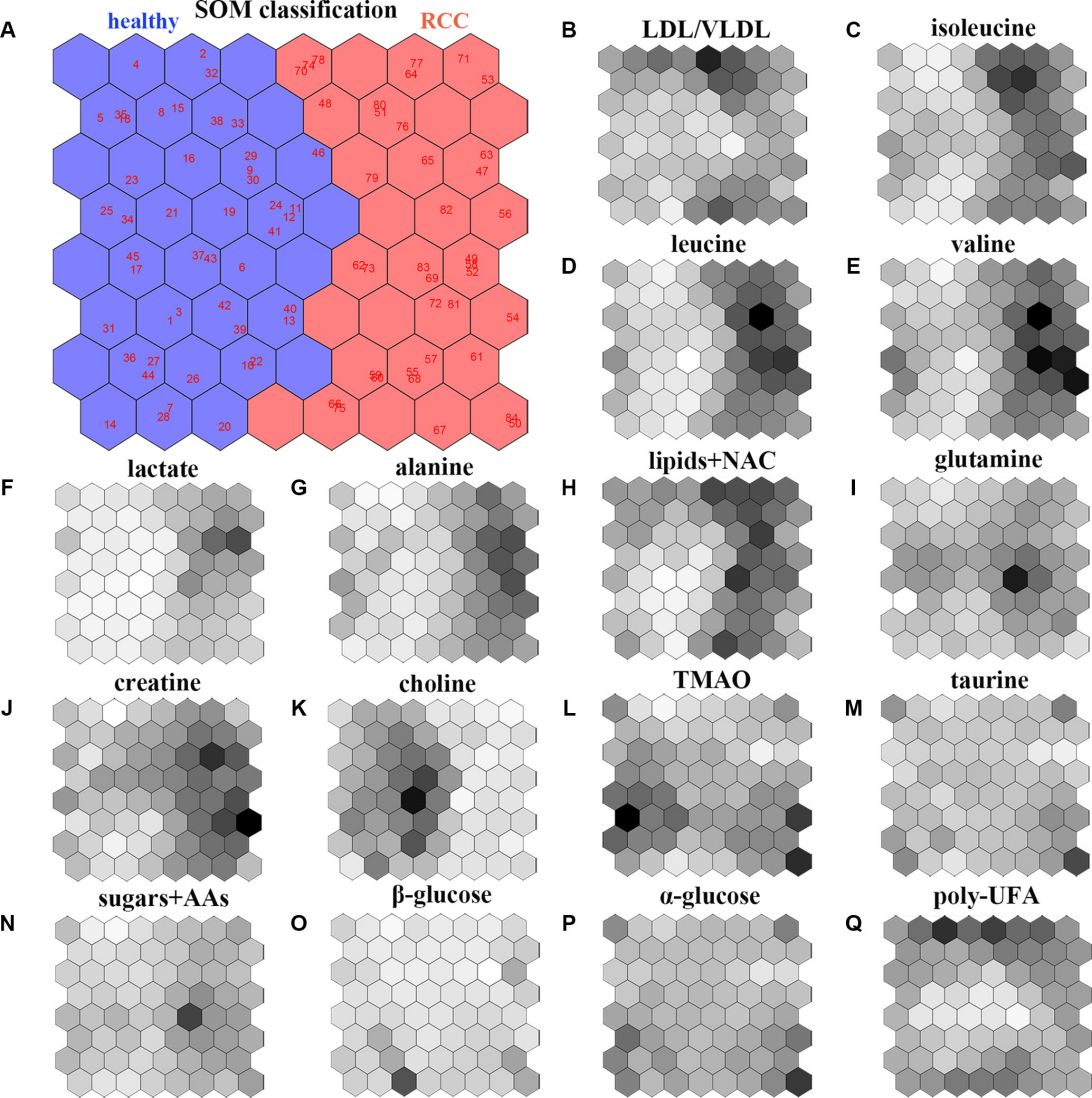
Analysis of the SOM model (**A**) Classification and prediction of healthy subjects and RCC patients using the SOM model based on all 16 metabolites obtained from the NMR-based serum metabolome: left region, healthy subjects; right region, early-stage RCC patients. The weight map for the 16 metabolites in the SOM model: (**B**) LDL/VLDL; (**C**) isoleucine; (**D**) leucine; (**E**) valine; (**F**) lactate; (**G**) alanine; (**H**) lipids+NAC; (**I**) glutamine; (**J**) creatine; (**K**) choline; (**L**) TMAO; (**M**) taurine; (**N**) sugars+AAs; (**O**) β-glucose; (**P**) α-glucose; (**Q**) poly-UFA. The deeper the color the higher the weight in the SOM.

**Figure 3:**
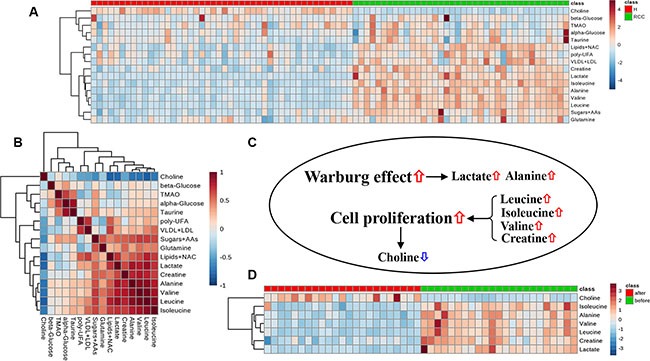
Metabolic data visualization Heatmap (**A**) and correlation (**B**) analyses of all 16 metabolites obtained from the NMR-based serum metabolome. Cluster analysis was performed using Ward's method and Euclidean distance. (**C**) Changes in metabolite levels in RCC patients and their biological effects in cancer cells. (**D**) Heat map analysis of seven metabolites as a biomarker cluster in RCC patients after nephrectomy.

The cluster of subjects that was generated based on the SOM (including all 16 metabolites and their respective weight maps) is shown in Figure 2. Healthy subjects and RCC patients were clustered on the left and right regions of the SOM, respectively (Figure 2A). Higher levels of very low density lipoprotein (VLDL)/low density lipoprotein (LDL), isoleucine, leucine, valine, lactate, alanine, lipids plus N-acetyl cysteine (NAC), and creatine were concentrated on the side of the RCC patients, while higher choline levels were observed on the side of the healthy subjects. However, the weight map for several metabolites including glutamine, trimethylamine N-oxide (TMAO), taurine, sugars plus amino acids (AAs), α-glucose, β-glucose, and poly-UFA, did not show a pattern similar to that of the cluster of subjects on the SOM shown in Figure 2A. Heat map analysis revealed that creatine, lactate, isoleucine, leucine, alanine, and valine clustered together (Figure 3A). The correlation map also demonstrated a strong positive relationship between these six metabolites (Figure 3B). Moreover, there was a strong negative correlation between choline and the six metabolites. Both alanine and lactate levels were increased in RCC patients, which was indicative of an enhanced Warburg effect in the cancer cells (Figure 3C). The increased creatine, isoleucine, leucine, and valine levels in RCC patients as well as the reduced choline levels could be responsible for cell proliferation. Importantly, following nephrectomy, RCC patients exhibited metabolic recovery as indicated by decreases in creatine, lactate, isoleucine, leucine, alanine, and valine levels as well as an increase in choline levels (Figure 3D). Therefore, these seven metabolites are specific for RCC and could be used as a biomarker cluster for the prediction and early diagnosis RCC.

### Prediction and diagnosis of RCC using the SOM model and serum metabolome

**Figure 4:**
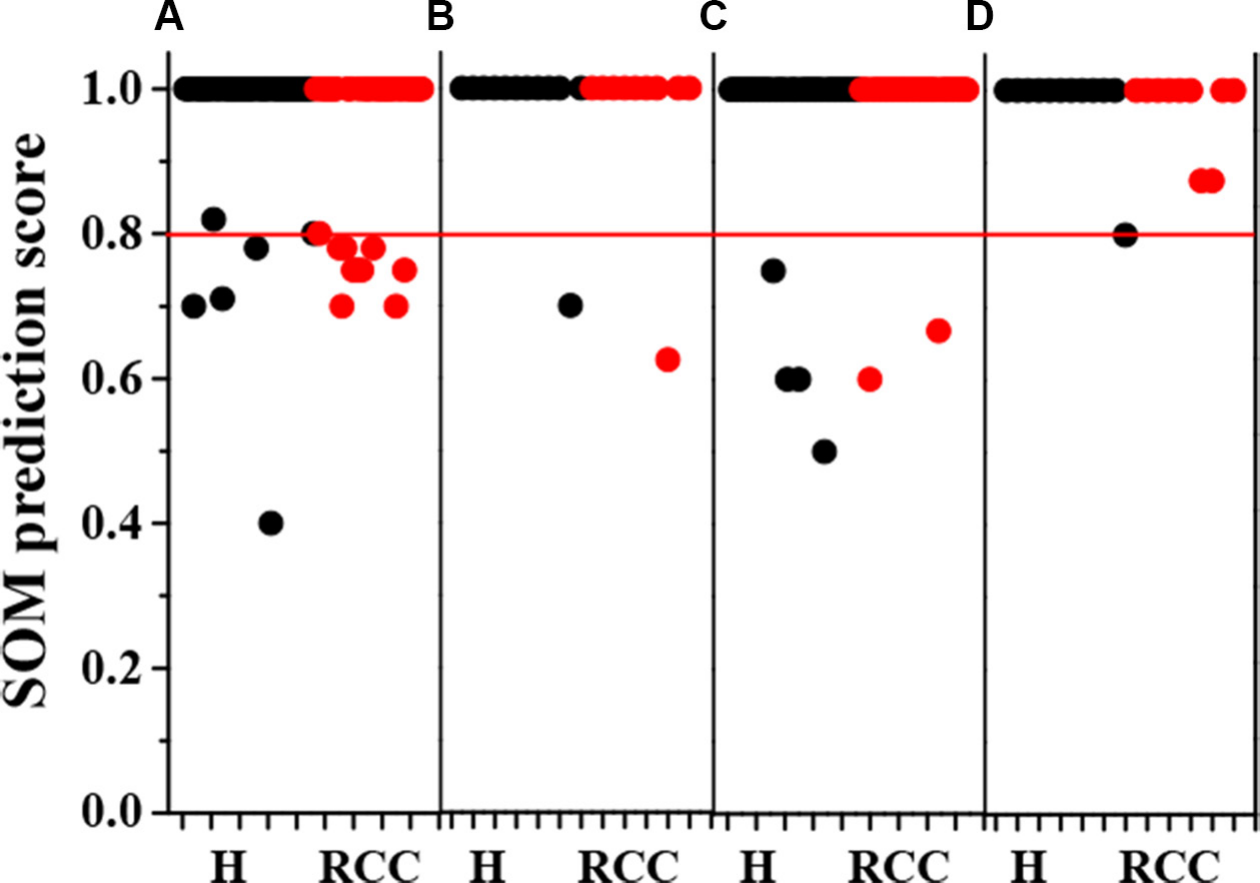
Accuracy of the SOM model in predicting early-stage RCC The SOM model based on all 16 metabolites: (**A**) training set; (**B**) test set. The SOM model based on the biomarker cluster: (**C**) training set; (**D**) test set. Black and red points represent healthy subjects (*N* = 46 in the training set; *N* = 12 in the test set) and RCC patients (*N* = 38 in the training set; *N* = 10 in the test set), respectively. Red line represents a cutoff value of 0.80 for RCC diagnosis, suggesting that the diagnosis was uncertain only if the prediction score was below 0.80.

The SOM (equipped with an optimal architecture) was trained on 80% of the subjects. The remaining 20% of the subjects were then used for validation of the trained SOM model. The predictive accuracy of the SOM model based on either all metabolites or the biomarker cluster is shown in Figure 4. A subject was classified into a category if the prediction score for the specific category was sufficiently high. In this study, we used a cutoff value of 0.80 to establish a diagnosis of RCC, which meant that if the SOM prediction score of a subject was below 0.80, the diagnosis was uncertain. Cut-off values are typically user-defined. We achieved a prediction accuracy of 93.48% for healthy subjects and 76.32% for RCC patients in the training set using all metabolites (Figure 4A). Using the biomarker cluster, we achieved a prediction accuracy of 91.30% in healthy subjects and 94.74% in RCC patients (Figure 4C). We next analyzed a set of 22 additional independent subjects in order to evaluate the predictive ability of the trained SOM model for RCC. There were two subjects who fell below the 0.80 prediction score in the test set when all metabolites were included (Figure 4B), while the trained SOM model using the biomarker cluster had higher predictive ability (Figure 4D). The detailed results for RCC prediction and diagnosis using the biomarker cluster are shown in Table 1. The results obtained using the SOM for the prediction and diagnosis of RCC were in agreement with the histological diagnosis for all subjects.

**Table 1:** Prediction and diagnosis of RCC using the SOM model and a biomarker cluster

Sample label	SOM prediction score		SOM prediction	SOM diagnosis	Histological diagnosis
	H^a^	RCC^b^			
P1	1.00	0.00	H	H	H
P2	1.00	0.00	H	H	H
P3	1.00	0.00	H	H	H
P4	1.00	0.00	H	H	H
P5	1.00	0.00	H	H	H
P6	1.00	0.00	H	H	H
P7	1.00	0.00	H	H	H
P8	1.00	0.00	H	H	H
P9	1.00	0.00	H	H	H
P10	1.00	0.00	H	H	H
P11	1.00	0.00	H	H	H
P12	0.80	0.20	H	H	H
P13	0.00	1.00	RCC	RCC	RCC
P14	0.00	1.00	RCC	RCC	RCC
P15	0.00	1.00	RCC	RCC	RCC
P16	0.00	1.00	RCC	RCC	RCC
P17	0.00	1.00	RCC	RCC	RCC
P18	0.00	1.00	RCC	RCC	RCC
P19	0.13	0.87	RCC	RCC	RCC
P20	0.13	0.87	RCC	RCC	RCC
P21	0.00	1.00	RCC	RCC	RCC
P22	0.00	1.00	RCC	RCC	RCC

a Healthy

b Renal cell carcinoma.

### Evaluation of metabolic patterns in RCC patients following nephrectomy

To evaluate changes in the metabolic patterns of RCC patients before and after nephrectomy, we analyzed a set of 23 RCC patients using the trained SOM model and biomarker cluster (Table 2). Prior to nephrectomy, all RCC patients were assigned to the RCC category, which was in agreement with the histological diagnoses. Interestingly, the trained SOM model using the biomarker cluster assigned all patients to the healthy group post-nephrectomy (Table 2). These data were indicative of metabolic recovery. However, the SOM prediction scores for three patients (A2, A5, and A11) were less than 0.80 (Table 2). Therefore, we could not definitively determine metabolic recovery in these patients. After 8 years, patient A5 suffered from renal failure, patient A11 died from RCC metastasis, and patient A2 was lost to follow-up. Follow-up was successful for 17 RCC patients 8 years post-nephrectomy, and 11 of the patients who displayed metabolic recovery remained healthy (Table 2).

**Table 2:** Prediction and diagnosis of RCC after nephrectomy using the SOM model and a biomarker cluster

Sample label	SOM prediction score		SOM prediction	SOM diagnosis	Histological diagnosis	8-year follow-up
	H^a^	RCC^b^				
B1	0.00	1.00	RCC	RCC	RCC	-^c^
B2	0.00	1.00	RCC	RCC	RCC	-
B3	0.00	1.00	RCC	RCC	RCC	-
B4	0.00	1.00	RCC	RCC	RCC	-
B5	0.00	1.00	RCC	RCC	RCC	-
B6	0.00	1.00	RCC	RCC	RCC	-
B7	0.00	1.00	RCC	RCC	RCC	-
B8	0.00	1.00	RCC	RCC	RCC	-
B9	0.00	1.00	RCC	RCC	RCC	-
B10	0.00	1.00	RCC	RCC	RCC	-
B11	0.00	1.00	RCC	RCC	RCC	-
B12	0.00	1.00	RCC	RCC	RCC	-
B13	0.00	1.00	RCC	RCC	RCC	-
B14	0.00	1.00	RCC	RCC	RCC	-
B15	0.00	1.00	RCC	RCC	RCC	-
B16	0.00	1.00	RCC	RCC	RCC	-
B17	0.00	1.00	RCC	RCC	RCC	-
B18	0.12	0.88	RCC	RCC	RCC	-
B19	0.00	1.00	RCC	RCC	RCC	-
B20	0.00	1.00	RCC	RCC	RCC	-
B21	0.00	1.00	RCC	RCC	RCC	-
B22	0.00	1.00	RCC	RCC	RCC	-
B23	0.00	1.00	RCC	RCC	RCC	-
A1	1.00	0.00	H	H	-	H
A2	0.75	0.25	H	-	-	LF^d^
A3	1.00	0.00	H	H	-	LF
A4	1.00	0.00	H	H	-	H
A5	0.67	0.33	H	-	-	RF^e^
A6	0.80	0.20	H	H	-	H
A7	1.00	0.00	H	H	-	DM^f^
A8	0.80	0.20	H	H	-	H
A9	1.00	0.00	H	H	-	H
A10	1.00	0.00	H	H	-	H
A11	0.75	0.25	H	-	-	DM
A12	1.00	0.00	H	H	-	LF
A13	1.00	0.00	H	H	-	H
A14	0.83	0.17	H	H	-	LF
A15	0.83	0.17	H	H	-	H
A16	1.00	0.00	H	H	-	H
A17	1.00	0.00	H	H	-	H
A18	1.00	0.00	H	H	-	H
A19	0.83	0.17	H	H	-	LF
A20	1.00	0.00	H	H	-	RF
A21	0.80	0.20	H	H	-	LF
A22	1.00	0.00	H	H	-	DM
A23	1.00	0.00	H	H	-	DM

a Healthy

b Renal cell carcinoma

c No data

d Lost to follow-up

e Renal failure

f Death from metastasis. A1-A23: RCC patients after nephrectomy; B1-B23: RCC patients before nephrectomy.

## DISCUSSION

Early diagnosis plays a key role in cancer treatment. However, the early diagnosis of RCC is challenging because it is non-palpable and patients are generally asymptomatic. The diagnosis of RCC is established using radiological examination, renal biopsy, and histologic analysis [16]. Omics-based strategies including genomics [17–19], proteomics [20–24], and metabolomics [12–14, 25] have the potential to assist RCC prediction and diagnosis. Biomarkers indicative of physiological changes between normal and disease states are important for omics-based approaches to RCC diagnosis and treatment [26–28]. Moreover, approaches based on multiple biomarkers have improved the robustness of cancer prediction and diagnosis compared to single biomarker approaches in clinical trials [29]. In this study, we identified a biomarker cluster comprised of alanine, choline, creatine, lactate, isoleucine, leucine, and valine for the prediction and early diagnosis of RCC.

The most fundamental metabolic change in cancer cells is an increase in aerobic glycolysis known as the Warburg effect [30]. In normal cells, glucose is first metabolized to pyruvate via glycolysis, which then enters the TCA cycle. However, in cancer cells, pyruvate is transformed to lactate or alanine instead of entering the TCA cycle, even under sufficient oxygen conditions. We found that RCC patients had higher levels of lactate and alanine in serum compared to healthy subjects. In addition, up-regulation of branched-chain AAs and creatine production in cancer cells can provide substrates for energy and protein synthesis, which are required for cell proliferation [31]. Since choline is involved in the synthesis of cellular membranes, a decrease in choline level may be attributed to cell proliferation. The levels of these metabolites were reversed in RCC patients after nephrectomy indicating that they are highly specific for RCC.

Here, we developed a SOM method based on a biomarker cluster of seven metabolites to predict and diagnose early-stage RCC. Our method could be used to identify early-stage RCC patients with 94.74% accuracy. To test the generalizability of the approach, a set of 22 additional independent subjects was analyzed. All of the subjects in this independent cohort were classified into the correct diagnostic categories. The goal of precision medicine is to determine ‘the right treatment, for the right patient, at the right time’ [32]. The method we proposed here can be used to accurately predict and diagnose early-stage RCC, thereby providing effective guidance for treatment. Although other linear methods combined with metabolomics-based approaches may also enable RCC classification [12–14], our method is advantageous because it easily accommodates both the linear and nonlinear features of metabolic information. Most importantly, the SOM was able to learn and store new knowledge from new datasets (constant updating) [33]. A non-invasive omics-based diagnosis using blood samples will be a promising diagnostic tool for early-stage RCC.

We also used the proposed method to evaluate changes in the metabolic patterns of RCC patients before and after nephrectomy. According to the SOM prediction, metabolic recovery to normal patterns occurred in all RCC patients after nephrectomy. Using a diagnostic criterion of 0.80, only 3 of 23 RCC patients were not confidently assessed as metabolically recovered. Therefore, our method is also an effective tool for evaluating postoperative metabolic status. Both gene [34, 35] and protein [36] expression data have been used to predict RCC patient survival. In this study, 17 patients were successfully followed-up 8 years post-nephrectomy. Eleven of the patients who exhibited metabolic recovery remained healthy while two patients with prediction scores less than 0.80 suffered from renal failure or died from RCC metastasis. Thus, our method may also be capable of predicting RCC patient survival.

To our knowledge, this is the first approach based on a combination of an SOM and a biomarker cluster identified using serum metabolomics data for RCC prediction and early diagnosis. Although the number of samples was limited and further clinical evaluation is necessary, we believe that our method can be used as a diagnostic tool for early-stage RCC. Ultimately, this method could enable RCC diagnosis using a simple blood test. We will use this approach in our hospital as a reference for RCC diagnosis to improve the robustness and accuracy. Additionally, we plan to build a RCC metabolite database and establish a standard procedure for the predictive diagnosis of RCC.

## MATERIALS AND METHODS

### Sample collection

Blood samples were collected from 68 healthy subjects and 58 patients with early RCC after clinical examination between 2006 and 2007. Participants who were not treated with any medications for the previous 3 months fasted for 12 h and then had blood drawn (approximately 5 mL) from the antecubital vein. Serum samples were separated following centrifugation at 1,024 g for 10 min at 4^°^C and stored at –80^°^C until NMR metabolomics analysis. RCC was diagnosed by a pathological investigation and graded according to the Union for International Cancer Control (UICC) tumor-node-metastasis (TNM) staging system [37]. RCC without metastases (T1–2, limited to the kidney) was categorized as early-stage. The characteristics of all participants are shown in Table 3. Serum samples from 23 RCC patients were also collected 6 months after nephrectomy in order to evaluate changes in metabolic patterns. In 2015, an 8-year follow-up was conducted by phone to examine RCC patient quality of life after nephrectomy. This study was approved by the Ethics Committee of Shanghai Jiao Tong University School of Medicine.

**Table 3:** Participant characteristics

Case	*N*^a^	TNM feature^b^	Gender (male)	Age (years)
Healthy	68	-	34	52.5 ± 15.1
RCC^c^ (without metastasis)	58	T1a (*N* = 20): < 4 cm, limited to the kidney	8	53.0 ± 11.5
		T1b (*N* = 20): 4–7 cm, limited to the kidney	10	52.3 ± 12.9
		T2 (*N* = 18): > 7 cm, limited to the kidney	12	58.7 ± 9.4
Nephrectomy	23	T1 (*N* = 13) T2 (*N* = 10)	18	53.3 ± 10.1

a Number of subjects

b Refer to Edge et al. [37]

c Renal cell carcinoma.

### NMR-based metabolomics analysis

Serum samples were thawed and vortexed, and 200 μL aliquots mixed with 400 μL of 0.2 M phosphate buffer to minimize pH variations. The mixture was centrifuged at 12,000 g for 10 min at 4°C, and 500 μL of the supernatant mixed with 100 μL D_2_O (field frequency lock) in a 5 mm NMR tube for NMR analysis. Proton NMR spectra were acquired at 25°C using a Varian Unity INOVA 600 NMR spectrometer with a triple resonance probe and z-axis pulsed field gradient (Bruker BioSpin, Rheinstetten, Germany). Standard one-dimensional (1D) PRESAT spectra were recorded using a single 90° pulse sequence and 1D spin-echo spectra acquired using the CPMG pulse sequence. The main acquisition parameters included: data points, 32 K; relaxation delay, 4 sec; spectral width, 10,000 Hz; acquisition time, 1.64 sec per scan; exponential line-broadening function, 0.3 Hz.

NMR spectra were preprocessed using phase and baseline corrections in the Topspin 3.0 software (Bruker BioSpin, Rheinstetten, Germany). NMR spectra were then referenced to the methyl signal of lactate at 1.33 ppm. The ‘icoshift’ procedure was performed to align all NMR spectra in MATLAB (R2012a, Mathworks Inc., Natick, MA, USA) [17, 38]. For dimensionality reduction, the spectral region from 0.4–10.0 ppm excluding the residual water signals from 4.4–5.2 ppm was subdivided and integrated to binned data with a size of 0.04 ppm. The NMR signals were carefully evaluated to exclude poorly aligned peaks and merged peaks derived from the same metabolites. The ^1^H NMR spectra from human serum samples are shown in Figure 1A. A total of 16 metabolite signals were prepared and assigned as shown in Table S1 based on previously reported data [39, 40] and the human metabolome database [41]. Two-dimensional ^1^H-^1^H COSY and TOCSY experiments for several representative samples were performed in order to confirm the assignments.

### SOM development

A SOM is a type of artificial neural network [33] that can learn from complex and high-dimensional data and project the information into a two-dimensional visual map. The SOM theory is derived from simulations of human brain function. For example, various sensory impressions have been mapped within the brain via neuronal systems. Here, a SOM was developed to predict and diagnose early-stage RCC based on serum metabolomics (Figure 1B). The procedure for SOM development is shown in Figure 1C. All data were auto-scaled and randomly divided into two subsets: a training set (80%) and validation set (20%). Next, genetic algorithms were used to optimize the most suitable the SOM architecture, which included the number of neurons and epochs [42] (Figure 1D). A hexagonal topology and cross-validation with venetian blinds (*n* = 10) were selected. The number of neurons and epochs was set from 4–16 and 50–400, respectively, for optimization. The optimal SOM architecture was then trained and validated using 80% and 20% of the subjects, respectively. An independent cohort consisting of 22 subjects was used to test the predictive capability of the trained SOM model for early RCC. Finally, postoperative changes in metabolic patterns before and after nephrectomy were evaluated in an additional cohort of 23 RCC patients. The SOM was developed using the Kohonen and CP-ANN toolbox [43] in MATLAB (R2012a, Mathworks Inc., Natick, MA, USA). In addition, heat map and correlation analyses were performed using MetaboAnalyst 3.0 [44].

## SUPPLEMENTARY MATERIALS TABLE


