# Supplementary material for: Prediction and diagnosis of renal cell carcinoma using nuclear magnetic resonance-based serum metabolomics and self-organizing maps
Source: Oncotarget. 2016 Jul 24;7(37):59189–98. doi: 10.18632/oncotarget.10830 (PMC5312304; doi:10.18632/oncotarget.10830)
Supplement: Supplementary file 1 [file oncotarget-07-59189-s001.pdf]

Prediction and diagnosis of renal cell carcinoma using nuclear magnetic resonance-based serum metabolomics and self-organizing maps

Supplementary Materials

Table S1: Assignment of metabolites in ^{1}H NMR spectra from human serum samples

No.	Compound	ppm	Assignment	Multiplicity
1	LDL/VLDL	0.84–0.91, 1.22–1.31	$CH_3(CH_2)_n/CH_3CH_2CH_2C$ =, $(CH_2)_n/CH_2CH_2CH_2CO$	m, m
2	isoleucine	0.92–0.93, 1.00–1.02	d-CH_3, b-CH_3	t, d
3	leucine	0.94–0.97	d-CH_3	d, d
4	valine	0.98–1.00, 1.03–1.05	$CH_{3,}$ CH_3	d, d
5	lactate	1.32–1.35, 4.10–4.15	CH_3, CH	d, q
6	alanine	1.47–1.50	CH_3	d
7	lipids + NAC	1.98–2.10	-	m
8	glutamine	2.42–2.48	half g-CH_2	m
9	creatine	3.04–3.05	CH_3	s
10	choline	3.20–3.22	$N(CH_3)_3$	s
11	TMAO	3.26–3.27	$N(CH_3)_3$	s
12	taurine	3.40–3.42	CH_2	t
13	sugars + AAs	3.48–3.88	various	m
14	β-glucose	4.63–4.67	CH	d
15	α-glucose	5.22–5.25	CH	d
16	poly-UFA	5.26–5.36	$CH = CHCH_2CH = CH$, = $CHCH_2CH_2$	m

Abbreviations: s, singlet; d, doublet; t, triplet; q, quartet; m, multiplet; LDL, low density lipid; VLDL, very low density lipid; NAC, *N*-acetyl signals from α_1-acid glycoproteins; TMAO, *N*-oxide trimethylamine; AA, amino acid.
